# HLA-Class II Artificial Antigen Presenting Cells in CD4^+^ T Cell-Based Immunotherapy

**DOI:** 10.3389/fimmu.2019.01081

**Published:** 2019-05-17

**Authors:** Alexandre Couture, Anthony Garnier, Fabian Docagne, Olivier Boyer, Denis Vivien, Brigitte Le-Mauff, Jean-Baptiste Latouche, Olivier Toutirais

**Affiliations:** ^1^UNIROUEN, Inserm U1245, Institute for Research and Innovation in Biomedicine, Normandie University, Rouen, France; ^2^Inserm U1237, Physiopathology and Imaging of Neurological Disorders, Caen University Hospital, Caen, France; ^3^Department of Immunology and Biotherapy, Inserm U1234, Institute for Research and Innovation in Biomedicine, UNIROUEN, Rouen University Hospital, Normandie University, Rouen, France; ^4^Department of Clinical Research, Caen University Hospital, Caen, France; ^5^Department of Immunology and Immunopathology, Caen University Hospital, Caen, France; ^6^Department of Genetics, Rouen University Hospital, Rouen, France; ^7^French Blood Service (Etablissement Français du Sang), Caen, France

**Keywords:** CD4^+^ T lymphocytes, adoptive cell therapy, cancer, autoimmunity, artificial antigen presenting cells, HLA class II molecules

## Abstract

CD4^+^ T cells differentiate into various T helper subsets characterized by distinct cytokine secreting profiles that confer them effector functions adapted to a variety of infectious or endogenous threats. Regulatory CD4^+^ T cells are another specialized subset that plays a fundamental role in the maintenance of immune tolerance to self-antigens. Manipulating effector or regulatory CD4^+^ T cells responses is a promising immunotherapy strategy for, respectively, chronical viral infections and cancer, or severe autoimmune diseases and transplantation. Adoptive cell therapy (ACT) is an emerging approach that necessitates defining robust and efficient methods for the *in vitro* expansion of antigen-specific T cells then infused into patients. To address this challenge, artificial antigen presenting cells (AAPCs) have been developed. They constitute a reliable and easily usable platform to stimulate and amplify antigen-specific CD4^+^ T cells. Here, we review the recent advances in understanding the functions of CD4^+^ T cells in immunity and in immune tolerance, and their use for ACT. We also describe the characteristics of different AAPC models and the way to improve their stimulating functions. Finally, we discuss the potential interest of these AAPCs, both as fundamental tools to decipher CD4^+^ T cell responses and as reagents to generate clinical grade antigen-specific CD4^+^ T cells for immunotherapy.

## Introduction

Functionally distinct CD4^+^ T cell subsets orchestrate immune responses against pathogenic microorganisms or transformed cells (1, 2). Each type of CD4^+^ T helper (Th) cells is endowed with a specific cytokine profile that regulates adaptive and innate immunity. Since a long time, it has been known that CD4^+^ T cells have a crucial role to support CD8 and B cell responses. CD4^+^ T cells also exert direct anti-tumor and anti-viral roles based on their cytolytic activity and effector cytokine secretion. Besides CD4^+^ effector cells, CD4^+^ regulatory T cells (Tregs), consisting of thymus-derived or induced cells, maintain peripheral tolerance to self-antigens by regulating other types of immune cells (3). In many autoimmune diseases, a defect in peripheral blood Treg number or immunosuppressive function has been described (4). Due to their multiple functions in immunity and immune tolerance, targeting CD4^+^ T cells has important clinical applications to treat cancer and chronic viral diseases, or to induce tolerance in autoimmune diseases and allograft. *In vivo* and/or *in vitro* approaches could be harnessed to develop CD4^+^ T cell-based immunotherapy. Several types of artificial antigen presenting cells (AAPCs) have been engineered through gene transfer allowing expression of presentation and costimulatory molecules required to stimulate antigen-specific CD4^+^ T cells. In this review, we describe our current understanding of CD4^+^ T cell functions in immunity and immune tolerance and discuss their contribution in adoptive cell therapy (ACT). We then focus on AAPCs as potent tools to induce specific CD4^+^ T cells *in vitro*. Finally, we examine the different ways to optimize AAPC models with the goals of reinforcing our basic knowledge of human CD4 responses and to propose efficient specific CD4^+^ T cell expansion protocols for ACT.

## Antigen Presentation By Mhc Class Ii Molecules and Cd4^+^ T Cell Activation

**Figure 1:**
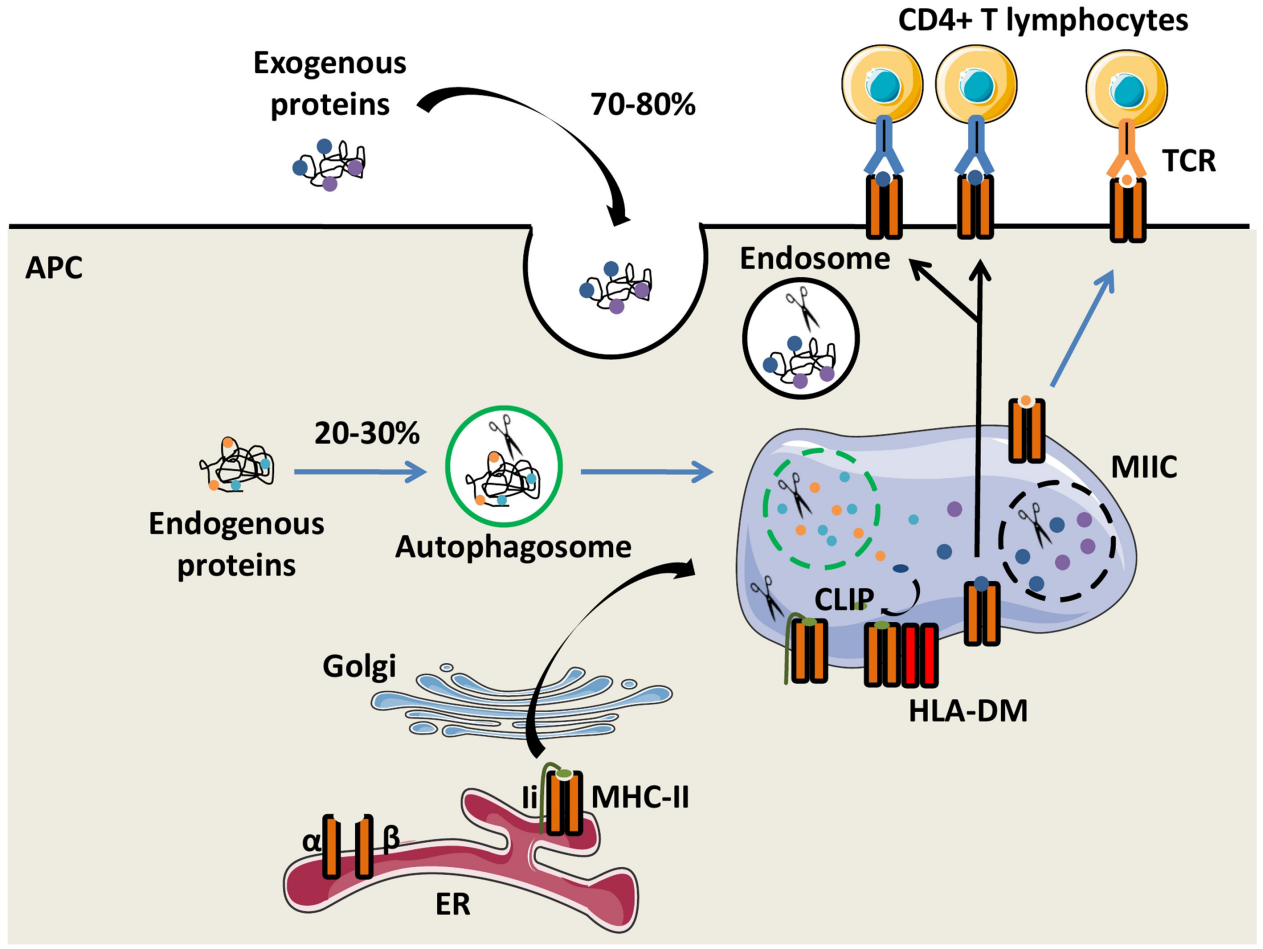
The MHC-II antigen presentation pathways. Major histocompatibility complex class II (MHC-II) α and β chains, expressed by antigen presenting cells (APCs), are synthetized in the endoplasmic reticulum (ER) where they form a heterotrimer with the invariant chain (Ii). After maturation in the Golgi apparatus, the heterotrimer (α/β/Ii) is delivered to the MHC class II compartment (MIIC) in which endocytosed and exogenous proteins but also Ii are degraded by proteases for generating peptides. Ii is progressively degraded into the Class II Invariant chain Peptide (CLIP) which binds to the MHC-II groove. The chaperone protein HLA-DM induces CLIP replacement by an antigenic peptide. Then, the peptide/MHC-II complexes move to the plasma membrane and are presented to T-cell receptors (TCRs) of CD4^+^ T lymphocytes.

The function of the major histocompatibility complex class II (MHC-II) molecules is to present antigens to CD4^+^ T cells. Their expression is preferentially restricted to professional antigen-presenting cells (APCs), including dendritic cells (DCs), monocytes/macrophages and B lymphocytes. In humans, HLA-DR, HLA-DQ, and HLA-DP molecules are the three classical and highly polymorphic MHC-II molecules. Both glycoproteins α and β chains of the MHC-II molecules are synthetized in the endoplasmic reticulum (ER) and are associated with the invariant chain (Ii), a chaperone molecule which stabilizes the conformation of the MHC dimer, avoiding the binding of endogenous peptides in the groove of MHC-II molecules. The heterotrimer is then transported out of the ER through the Golgi apparatus to a specialized endosome, the MHC-II compartment (MIIC), in which proteases, such as cathepsins degrade Ii. Only a part of Ii, the class II invariant chain peptide (CLIP), is maintained in the MHC-II groove (5). The non-classical MHC-II molecule HLA-DM interacts with MHC-II molecules and catalyses the exchange of CLIP with a high-affinity peptide. Action of HLA-DM is inhibited by another non-classical MHC-II molecule, HLA-DO (6). Peptides not covalently binding the MHC-II molecules are mainly derived from exogenous self-and non-self-proteins degraded by the endocytic pathway. Of note, a substantial fraction of MHC-II-associated peptides comes from the cytosolic proteins through the autophagic pathway (7). Then, peptide-MHC-II (pMHC-II) complexes are transferred and displayed on the plasma membrane of APCs for recognition by CD4^+^ T cells (Figure 1).

**Figure 2:**
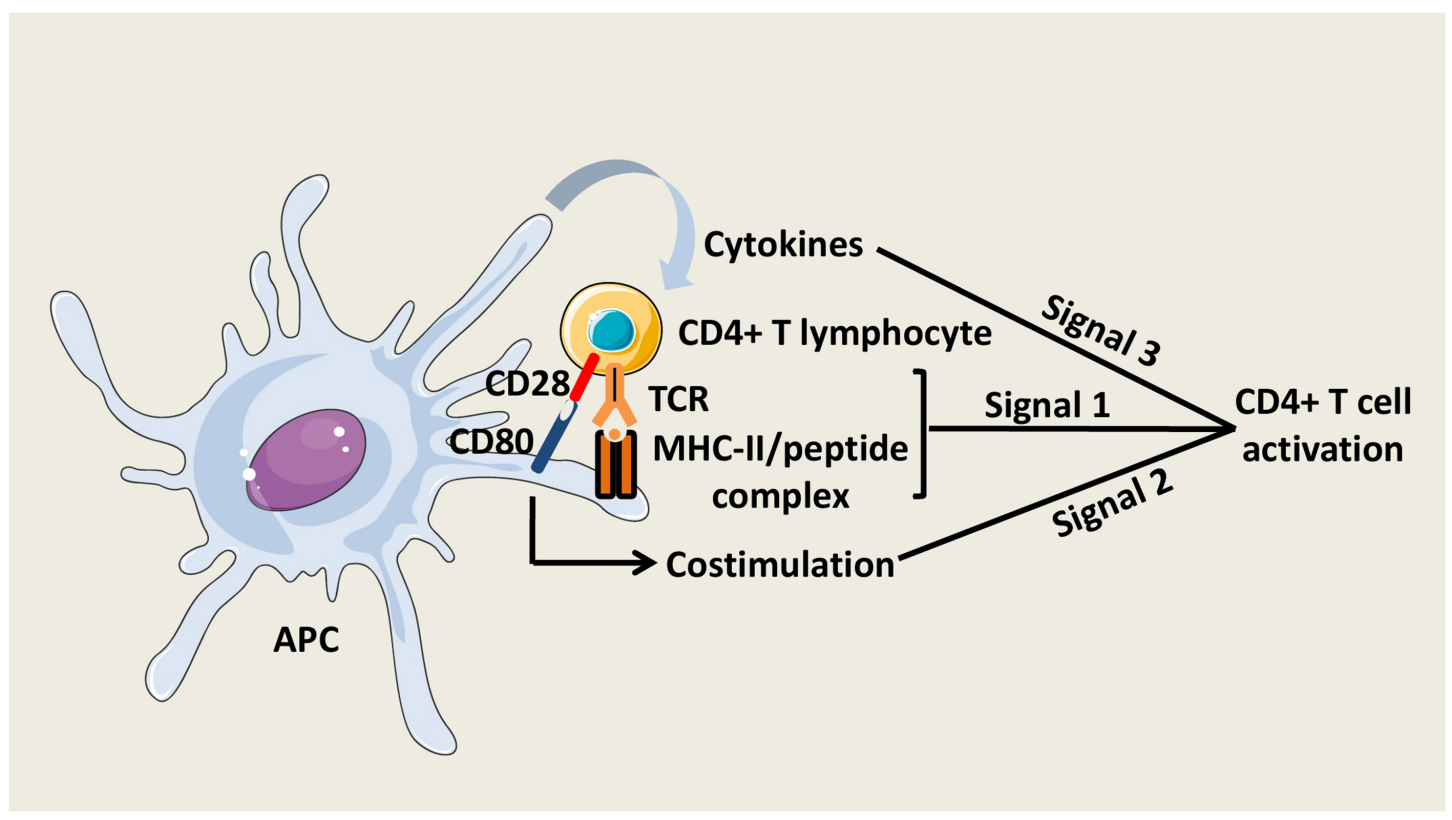
CD4^+^ T cell activation. Three signals are necessary to fully activate CD4^+^ T cells. The first signal is mediated by the interaction between TCRs and peptide/MHC-II complexes at the APC surface. The second signal is mediated by costimulatory molecules, such as CD80 on APCs, which interacts with CD28 on CD4^+^ T cells. The third signal is delivered by soluble factors including cytokines, which are notably secreted by the APCs.

The activation of naïve CD4^+^ T cells is initiated by the interaction of T Cell Receptors (TCRs) with specific pMHC-II complexes presented by professional APCs. Close contact between T cell and APC leads to the formation of a specialized structure named the immunological synapse (8). To fully prime CD4^+^ T cells, antigenic signal is reinforced by costimulatory molecule interactions with APCs and by cytokines secreted in the local environment (Figure 2). The main costimulatory molecule expressed by T cells is CD28, which interacts with CD80 and CD86 on APCs. CD40 molecule on APCs that binds CD40L on activated T cells is also critical for CD4^+^ T cell responses, at least, in part, by amplifying APC activation.

## Effector Cd4^+^ T Subsets

CD4^+^ T cells differentiate into diverse effector T helper (Th) subsets producing characteristic cytokines adapted to their functions and providing to the host the capacity to develop optimal immune responses against different types of pathogens (Table 1) (2, 9–13). Polarization of Th cells is governed by distinct transcription factors induced by cytokine environments. The stability of polarized CD4^+^ T cell subsets is still debated since many extracellular and cytosolic signals may contribute to their complex plasticity (14).

**Table 1:** Main features of CD4^+^ TL subsets arising from naïve CD4^+^ T cells.

**CD4^+^ T lymphocyte subsets**	**Cytokines involved in T cell differentiation**	**Transcription factors**	**Cytokines and soluble factors**	**Functions**	**Roles in immunopathology**
Th1	IFN-γ, IL-12	T-bet	IFN-γ, TNF-α	Immunity against intracellular pathogens (viruses, bacteria), cancer immunosurveillance	Delayed-type hypersensitivity
Th2	IL-4	GATA3	IL-4, IL-5, IL-13	Immunity against parasites, IgE production by B cells	Allergy
Th9	TGF-β, IL-4	PU.1, Foxo1	IL-9	Immunity against parasites, mucus secretion, cancer immunosurveillance	Inflammation
Th17	TGF-β, IL-6, IL-23	RORγt	IL-17A, IL-17F, IL-21, IL-22	Inflammation, immunity against extracellular pathogens, tissue homeostasis, cancer immunosurveillance (controversial)	Autoimmunity, inflammation
CD4 CTL	IL-2, IL-15	Eomes	Granzyme B, Perforin	Cytotoxicity, cancer immunosurveillance	Autoimmunity
Tfh	IL-6, IL-21	Bcl6	IL-21	B cell help	Autoimmunity
iTreg	TGF-β, IL-2	Foxp3	TGF-β, IL-10	Immune tolerance, immune suppression	Inhibition of cancer immunosurveillance

*Th, helper T cell; CTL, cytotoxic T lymphocyte; Tfh, follicular helper T cell; iTreg, induced regulatory T cell; IFN-γ, interferon-gamma; IL, interleukin; TGF-β, tumor growth factor-beta; T-bet, T-box expressed in T cells; GATA3, GATA binding protein 3; RORγt, retinoic acid receptor-related orphan receptor gamma thymus-restricted isoform; Eomes, Eomesodermin; Bcl6, B-cell lymphoma 6; Foxp3, Forkhead box P3; Foxo1, Forkhead box O1; TNF-α, tumor necrosis factor-alpha*.

Th1 cells that produce large amounts of IFN-γ and express T-bet are involved in the defense against intracellular pathogens. They activate macrophages and help the generation of effector and memory CD8^+^ T cells (2). The response against extracellular parasites is controlled by IL-4-producing Th2 cells that stimulate IgE production by B cells and activate eosinophils. Th2 cells have a crucial role in the pathogenesis of many allergic diseases (11).

Th17 cells secrete IL-17, IL-22, and GM-CSF, and have a high degree of plasticity (9, 10). They contribute to immunity against fungi and extracellular bacteria, mainly by inducing the release of anti-microbial peptides and attracting myeloid cells to infected mucosal surfaces, especially gut and skin. These cells are also involved in microbiota maintenance and epithelium integrity and regeneration, and promote humoral response in the gut (15). However, data from animal models and patients indicate a pathogenic role of Th17 cells in chronic inflammatory and autoimmune diseases, such as multiple sclerosis, psoriasis or systemic lupus erythematosus (16–18). Conversion of self-reactive effector Th17 cells into Th1-like Th17 cells producing IFN-γ and/or GM-CSF is a predominant mechanism of their pathogenicity identified in experimental autoimmune encephalomyelitis (EAE), a mouse model of multiple sclerosis (19, 20).

T follicular helper (Tfh) cells are involved in B cell help and humoral response against extracellular antigens, supporting germinal center formation and antibody production. Th9 cells that are mainly characterized by the secretion of IL-9, have protective roles against parasite infections and tumor development (21). Th9 promote worm clearance by inducing mucus secretion by epithelial cells. Th9 have a skin tropism but their functions in cutaneous immunity and skin disorders are currently under investigation (22–24). PU.1 is one of the major transcription factors associated with Th9 cells but recently another transcription factor, Foxo1, has been shown to be essential in their development (25). Detrimental functions of Th9 are also described in inflammatory and autoimmune diseases (26). For instance, IL-9 impairs intestinal tissue repair and exacerbates the disease in a mouse colitis model (27).

Another population recently described is the CD4^+^ T cells that harbor cytolytic activity against MHC-II^+^ target cells. Cell killing mechanisms involve perforin/granzyme and Fas-FasL pathways. *In vivo*, it was shown in the EAE model that Eomesodermin (Eomes) transcription factor drives the development of CD4^+^ cytotoxic T lymphocytes (CTLs) (28).

## Regulatory Cd4^+^ T Cell Subsets

CD4^+^ regulatory T cells (Tregs) are critical for maintaining peripheral tolerance and preventing autoimmunity. Depending on the expression of the transcription factor Forkhead box protein 3 (Foxp3), Tregs may be divided into two subsets: the classical Foxp3^+^ Tregs and the Foxp3-negative type 1 regulatory T (Tr1) cells. Foxp3^+^ Tregs can be generated in the thymus (thymus-derived Treg cells, tTregs) or also be induced from naïve T cells in the periphery (peripherally-induced Treg cells, pTregs) (29). A third subtype of Foxp3^+^ Tregs, termed iTregs, represents the *in vitro*-induced Tregs. Tr1 cells induced in the periphery, secrete IL-10 and/or transforming growth factor beta (TGF-β). Distinct intracellular and surface markers help distinguishing Foxp3^+^ Tregs and Tr1 cells. In addition to the expression of their specific transcription factor, Foxp3^+^ Tregs are identified by their constitutively high expression of cell surface IL-2Rα chain (CD25) in the absence of the IL-7Rα chain (CD127). Recently, coexpression of the lymphocyte-activation gene 3 (LAG-3) molecule and the integrin alpha-2 subunit (CD49b) was shown to characterize Tr1 cells (30). Tregs have a potent suppressive capacity toward a very broad range of immune cells. Foxp3^+^ Tregs and Tr1 cells share common mechanisms of immunosuppression involving inhibitory cytokine production, metabolic disruption *via* the expression of ectoenzymes, cytotoxic activity and inhibition of APCs (31). The potential of Treg-based immunotherapies in preventing autoimmune diseases or controlling graft vs. host disease (GVHD) and allograft rejection is attested by several studies in preclinical models (32–34). In these contexts, Treg-based therapeutic strategies rely on the *in vitro* or *in vivo* activation of natural or induced Tregs. They include adoptive transfer of Tregs and vaccination with autoantigen-derived peptides or other pharmalogical approaches (see below) (35, 36).

## Role of Cd4^+^ T Cells in Anti-Tumor and Anti-Viral Adaptive responses

Growing evidences in the literature indicate that CD4^+^ T cells have direct roles in anti-tumor and anti-viral responses without contribution of CD8 or B cells. Several effector mechanisms have been described depending on the experimental models and the investigated Th subsets. Quezada et al. have demonstrated that transfer of tumor-specific CD4^+^ cells in lymphopenic mice resulted in rejection of melanoma tumors (37). In this study, CD4^+^ T cells had a Th1-like phenotype, produced granzyme B and displayed a MHC class II-dependent cytotoxic activity. In another mouse adoptive transfer model, Th17-polarized T cells were also capable of rejecting melanoma tumors *via* an IFN-γ dependent mechanism (38). Nevertheless, Th17 cells can also have a protumor effect by inducing angiogenic factors (39).

More recently, several studies highlighted anti-tumor properties of IL-9 producing CD4^+^ T cells (40). Purwar et al. have found in the B16 melanoma mouse model that tumor growth was accelerated in IL-9 receptor-deficient mice while injection of recombinant IL-9 prevented tumor progression in wild-type mice (41). Other studies reported that anti-cancer effects of Th9 cells were mediated by production of IL-21 and their cytolytic activity (42).

CD8^+^ T cells are considered as the main effector cells of cancer and virus immunosurveillance, capable of killing tumors or infected cells and secreting immunostimulatory cytokines. Nevertheless, CD4^+^ T cell help is critical for maintaining CD8^+^ T cell functions during anti-tumor response and chronic infection (2, 43, 44). Indeed, CD4^+^ T cells are required to fully activate and “license” DCs which can effectively prime CD8^+^ T cells. CD40L-CD40 interactions between activated CD4^+^ T cells and DCs, respectively, are crucial to increase DC antigen-presentation and costimulation capacities (45). However, primary CD8^+^ T cell responses could be induced in a T cell help independent manner by microbial pathogen infections that provide potent inflammatory stimuli. Additionally, cognate interactions between activated CD4^+^ T cells and DCs lead to the production of chemokines that facilitate the recruitment of naïve CD8^+^ T cells toward antigen-bearing APCs in the secondary lymphoid organs (46). Although there is a consensus on the requirement of T cell help for the generation of long-lived memory CD8^+^ T cells, it is still discussed whether CD4^+^ T cells deliver a differentiation program during the priming phase or subsequently at later stages during the CD8^+^ T cell memory maintenance (47–49). Production of IL-2 by Th cells during the priming phase is crucial for an effective secondary CD8^+^ response (50). However, it has been shown that “licensed” DCs may provide signals that enable autocrine secretion of IL-2 by memory CD8^+^ T cells (51). CD4^+^ helper T cells also stimulate IL-15 production by DCs that favors induction of long-lived CD8^+^ T cells by increasing expression of anti-apoptotic molecule Bcl-xL (52). In the context of viral chronic infection, IL-21 is an essential component of CD4^+^ T cell help by avoiding clonal deletion and maintaining CD8^+^ T cell function (53). Regulation of activation-induced cell death (AICD) by CD4^+^ T cells is a putative mechanism for the maintenance of CD8^+^ T cell response. It has been reported that CD8^+^ T cells primed in the absence of CD4^+^ T cells could undergo AICD-mediated by TNF-related apoptosis-inducing ligand (TRAIL) signaling (54). However, other studies using TRAIL-deficient mice, do not confirm this mechanism (55). Furthermore, recently, CD4^+^ T cells have been shown to upregulate the expression of CD70 on DCs, a costimulatory molecule that triggers CD27 receptor on CD8^+^ T cells. CD70-CD27 interactions result in the delivery of a help program that amplifies CTL functions and downregulates inhibitory receptors, such as PD-1 (56).

Neutralizing antibodies are a central component of adaptive immune responses against microbial pathogens. In the previous decade, Tfh cells characterized by the expression of CXCR5 have been identified as the main providers of B cell help. CD40L on Tfh cells is the most prominent costimulatory molecule to promote survival and proliferation of CD40-expressing B cells. Cytokines IL-4 and IL-21 are necessary for the formation of germinal centers and differentiation of B cells into long-lived plasma cells (57). Recently the role of Tfh in anti-tumor immunity was underlined by several reports. In colorectal cancer, gene expression and tissue microarray analyses of tumor biopsies showed that tumor infiltrating Tfh and B cells were correlated with patient disease-free survival (58). The authors also found that chemokine CXCL13 and IL-21 were key factors of adaptive immunity in tumor environment. Infiltration of CXCL13-producing CD4^+^ Tfh cells was also associated with a better disease-free survival or preoperative response to chemotherapy (59).

## CD4^+^ T Cell-Based Immunotherapy

ACT clinical trials mainly focus on CD8^+^ effector cells and very few studies have investigated the therapeutic potential of CD4^+^ T cells. One of the first CD4^+^ T cell-based immunotherapy approaches was based on the infusion of an autologous CD4^+^ T-cell clone. Hunder et al. had expanded *in vitro*, a New York Esophageal Squamous Cell Carcinoma 1 (NY-ESO-1)-specific CD4^+^ T-cell clone from a refractory metastatic melanoma patient. After one infusion of a few billion cells, the patient presented a durable clinical remission (60). Another clinical trial has been conducted with autologous CD4^+^ T cells from patients transduced with a melanoma-associated antigen 3 (MAGE-3)-specific MHC-II-restricted TCR transgene (61). In a cohort of 17 patients with metastatic solid cancer and treated with 10^7^-10^11^ cells, one patient had a complete response and a partial response was observed in three patients.

With the breakthrough of next generation sequencing (NGS) technologies, it is now possible to characterize mutations within patient's tumor and finally identify potential immunogenic neoantigens in many cancers, such as melanoma or colon cancer (62–64). These findings could be translated into clinical applications. Tran et al. have identified Th1 cells specific for a neoantigen derived from the erbb2 interacting protein (ERBB2IP) in a metastatic cholangiosarcoma patient's tumor (65). Two infusions of several billion neoantigen-specific CD4^+^ tumor infiltrating lymphocytes led to a disease stabilization for more than 1 year. Ott et al. have identified neoantigens in six advanced melanoma patients and vaccinated them with a pool of synthetic long-peptides derived from the predicted neoantigens (66). No recurrence 25 months after treatment was observed in four patients.

A major problem associated with hematopoietic stem cell transplantation (HSCT), is *Epstein-Barr Virus* (EBV) or *cytomegalovirus* (CMV) reactivation, as well as opportunistic virus, such as adenovirus infections which are fatal in 20% of cases (67, 68). To avoid these complications, *in vitro* protocols have been developed to rapidly generate CD4^+^ and CD8^+^ multivirus-specific T cells by a single stimulation of donor peripheral blood mononuclear cells (PBMCs) with a pool of synthetic peptides covering the target viral antigens (69–71). In a recent clinical trial including 37 patients with viral infection after allogeneic HSCT, 92% had a complete or partial response after one infusion of 10^7^ virus-specific T cells (72).

## Regulatory T Cell-Based Immunotherapy

Compelling experimental data from mouse models indicate that adoptive immunotherapy harnessing immunosuppressive properties of CD4^+^ regulatory T cells is a promising therapeutic strategy against autoimmune diseases and GVHD or allograft rejection. This type of strategy has the advantages of allowing the accurate definition of the phenotype and functions of the infused cells and of avoiding the toxicity of general immunosuppressive drugs. Human peripheral blood CD4^+^ CD25^+^ Tregs, potentially including tTregs and pTregs, are the main source for such a cell therapy but they constitute only 1–2% of human CD4^+^ T cells. Due to the low number of circulating Tregs, translating Treg transfer approaches from mice studies to humans requires efficient protocols to generate large numbers of highly purified Tregs. Usually, cell therapy protocols consist in a first step of CD4^+^ CD25^+^ polyclonal Treg isolation using either magnetic bead-or flow cytometry-based systems. Next, isolated Tregs are either directly infused into patients or stimulated *in vitro* with anti-CD3/anti-CD28-coated beads (73).

So far, the therapeutic potential of polyclonal Tregs has been investigated in the context of GVHD and type 1 diabetes, with some evidences of safety and clinical benefits (74, 75). A possible drawback of using polyclonal Tregs in immunotherapy is the risk of inducing an overall immune suppression compromising beneficial immune responses. Autoantigen-specific Tregs may represent a better alternative for the treatment of autoimmune diseases, conferring a more localized and targeted immunosuppression at the site of inflammation. Noteworthy, several reports highlighted that antigen-specific Tregs were significantly more efficient than polyclonal Tregs in regulating autoimmune or allogenic responses in animal models (35, 76). To generate antigen-specific Tregs, polyclonal CD4^+^ CD25^+^ T cells could be stimulated with antigen-loaded APCs or transduced with a viral vector to express a TCR that recognizes a specific peptide. More recently, chimeric antigen receptors (CARs) designed to redirect human Tregs toward donor-MHC class I HLA-A2 molecule have been successfully used to prevent rejection in a skin xenograft transplant model (77, 78).

Additional immunoregulatory strategies that have already proven clinical efficacy could be combined with adoptive Treg immunotherapy to maximize a tolerogenic response in patients. IL-2 signaling is indispensable for Treg development and functions (79). Data from murine models and clinical trials in autoimmune diseases and HSCT have clearly shown that low-dose IL-2 administration led to increased numbers of Tregs *in vivo* and to beneficial therapeutic effects (80–83). Moreover, several immunosuppressive drugs, such as rapamycin, or immunomodulatory treatments including intravenous immunoglobulins could efficiently boost Treg response *in vivo* (84–86).

Mesenchymal stromal cells (MSCs) represent another major immune regulatory population currently assessed in cell therapy. MSCs inhibit T cell proliferation and DC maturation and also promote the expansion of Tregs (87–89). Therapeutic effects of MSCs have been reported in various autoimmune diseases, such as multiple sclerosis and diabetes, as well as in GVHD (90–92).

## Hla Class Ii-Aapcs: an Attractive Tool to Activate and Expand Antigen-Specific Cd4^+^ T Cells *In vitro*

Beads coated with anti-CD3 and anti-CD28 antibodies are commonly used to amplify polyclonal human effector T cells or Tregs *in vitro* (93, 94). The development of protocols to efficiently expand human antigen-specific CD4^+^ T cells is utterly needed and requires the availability of potent APCs. As mentioned above, an interesting approach to generate specific CD4^+^ T cells consist in priming autologous PBMCs loaded with peptides. This approach that is mostly appropriated for highly immunogenic antigens, usually viral antigens, may involve a direct selection step, such as the one based on magnetic bead-enrichment of IFN-γ-secreting T cells (69–71, 95). *In vivo*, DCs are the most efficient professional APCs to trigger functional adaptive T cell immunity. Indeed, it is well-established that DCs, mainly arising from monocytes, can elicit robust specific CD4^+^ T cell responses *in vitro* (96, 97). Nevertheless, the use of DCs has limitations. Their production *in vitro* is difficult to standardize in terms of cell number, phenotype and functionality (98). In addition, it requires supplementary blood from healthy donors or patients. In this context, AAPCs are an interesting alternative because they constitute cell lines especially engineered for more easily and reproducibly amplifying functional antigen-specific T cells. AAPCs have the advantages of being quickly available and suitable for any healthy donor or patient in a given HLA context.

**Figure 3:**
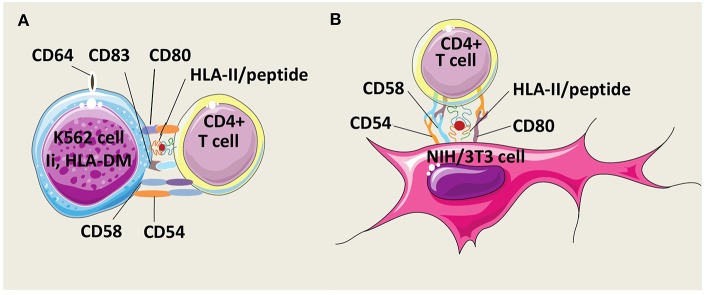
Models of HLA-II-AAPCs for the expansion of human antigen-specific CD4^+^ T cells. **(A)** Model of artificial antigen presenting cells (AAPCs) derived from erythroleukemic K562 cell line. K562-AAPCs express the invariant chain (Ii), HLA-DM, CD54, CD58, CD64, CD80, CD83, and one HLA class II (HLA-II) molecule. **(B)** Model of AAPCs derived from the mouse fibroblast NIH/3T3 cell line. NIH/3T3-AAPCs express CD54, CD58, CD80, and one HLA-II molecule.

Butler and Hirano have developed an AAPC model derived from the human erythroleukemic cell line K562 which constitutively expresses the adhesion molecules CD54 (intercellular adhesion molecule-1, ICAM-1) and CD58 (leukocyte function-associated antigen-3, LFA-3) but not HLA class I and II molecules (Figure 3A) (99). To mount human CD4^+^ T cell responses, K562 cells were genetically modified to express costimulatory molecules CD80 and CD83, and a single HLA-DR allele. In addition, K562 cells were equipped with Ii and HLA-DM molecules to foster antigen processing and presentation. After multiple stimulations, these AAPCs exogenously loaded with peptides were able to expand Th1 central memory (CD45RO^+^, CD62L^+^, CCR7^+^) antigen-specific CD4^+^ T cells against infectious antigens without growth of Treg cells. Unfortunately, no comparison data are available regarding specific T cell activation capacities of these AAPCs vs. autologous APCs. Spontaneously, K562 cells have a low ability to take up exogenous proteins. Transduction with CD64, a high affinity Immunoglobulin G (IgG) Fragment crystallizable (Fc) receptor (Fc gamma RI), improved uptake and presentation of CMV pp65, a whole protein antigen, under immune complex forms. K562-derived AAPCs have also been used to stimulate CD4^+^ T cells against Survivin, a tumor associated antigen (TAA) (100). However, TAAs are inherently poorly immunogenic and CD4^+^ T cells needed to be initially sensitized with antigen-pulsed autologous monocytes before restimulation with K562-derived AAPCs. Interestingly, CD4^+^ T cells were long-lived effector cells able to secrete IFN-γ (Th1) or IL-4 (Th2) after challenge with MHC-II-positive tumor cells.

Our group has designed another AAPC system using the mouse fibroblast cell line NIH/3T3 that has been retrovirally transduced to express a single HLA class II molecule, the costimulatory molecule CD80, and two adhesion molecules, CD54 and CD58 (Figure 3B). Previously, NIH/3T3 cells have been used to generate CTL responses after gene transfer with vectors encoding a single HLA class I molecule and an antigen in the form of a peptide or of a whole protein (101–103). These AAPCs consistently elicit strong stimulation and expansion of anti-tumor or anti-viral CD8^+^ CTLs. Thereafter, using the same murine fibroblast backbone, novel AAPCs expressing a single HLA class II molecule have been constructed to attempt to mount CD4^+^ T cell responses (104). We have demonstrated that these peptide-loaded AAPCs strongly activated specific CD4^+^ T-cell clones. In addition, they were able to efficiently and spontaneously take up, process extracellular whole proteins and present the correct epitope to specific T cells. In primary *in vitro* stimulation, AAPCs enabled expansion of antigen-specific CD4^+^ T cells, but with a lower stimulating ability than autologous APCs. Importantly, a major advantage of these AAPCs is their higher ability than autologous APCs to reactivate and amplify a high number of specific memory CD4^+^ T cells, at a scale compatible with therapeutic applications. Expanded T cells harbored transitional memory (CD45RO^+^, CD62L^+^, CCR7^−^) and effector memory (CD45RO^+^, CD62L^−^, CCR7^−^) phenotypes, and were highly functional Th1 cells that secreted IFN-γ upon antigen-specific stimulation.

## Optimization Of Aapc Systems and their Use Perspectives

We and others have shown that AAPCs constitute reliable systems to expand human CD4^+^ T cells (104, 105). Since AAPC models are easily customizable, they could be used as a tool for basic and/or applied research on CD4^+^ T cell responses. As a key step, antigen presentation could be optimized by genetic approaches to address antigens into the various cell compartments (cytoplasm, ER, endosome) involved in the HLA class II presentation pathway. Indeed, endogenous antigen expression in the form of peptide or whole protein might foster their presentation as it was observed in HLA class I-AAPCs (101, 103). In addition, these antigen-targeting strategies could also be exploited to dissect the alternative MHC-II pathway which remains poorly understood (106, 107).

Manipulating expression of different costimulatory molecules on AAPCs is another opportunity to increase T cell functions *in vitro*. The tumor necrosis factor receptor superfamily (TNFRSF) molecules CD27 and 4-1BB (CD137) are among the costimulatory molecules that appear to be important to enhance CD4^+^ T cell response. CD27 is expressed on naïve and memory T cells and interactions with its ligand CD70 on APCs promote both Th1 differentiation via IL-12Rβ2 up-regulation and survival of CD4^+^ T cells (108). Similarly, 4-1BB ligand on APCs enhance proliferation and effector functions of activated CD4^+^ T cells that express 4-1BB (109, 110). Co-signaling receptors also modulate differentiation of Tregs. Interestingly, programmed-death-ligand 1 (PD-L1) binding to programmed death 1 (PD-1), expressed by T cells, has been shown to induce the conversion of naïve CD4^+^ CD25^−^ T cells into iTregs in mouse models (111, 112). PD-L1 expressing AAPCs could be a useful tool to study the role of this co-inhibitory molecule in a human context.

Cytokines are critical parameters to promote the differentiation of specific CD4^+^ T cell subsets and to sustain their expansion *in vitro*. To date, culture protocols with AAPCs include IL-2 as a T cell growth factor and enable expansion of T cells with Th1 or Th2 phenotype (100, 104). Several studies have documented the cytokine requirement for the differentiation of Th subsets upon polyclonal stimulation. For instance, the cytokine mixture TGF-β1, IL-1β, IL-6, and IL-23 is known to promote Th17 differentiation whereas TGF-β1 and IL-4 enable generation of Th9 cells (113, 114). Whether AAPCs with appropriate cytokine cocktails could induce antigen-specific CD4^+^ T cell subsets remains to be examined. An important challenge for ACT is to generate T cells that persist *in vivo* and maintain long-term proliferative and functional abilities. The complex role of cytokines in CD4^+^ T cell responses is not fully understood. Among them, IL-7, IL-15 as well as IL-6 and IL-21 are involved in CD4^+^ T cell proliferation and survival, and AAPC systems should allow to better define their functions (115–120).

In clinical immunology and for research purposes, AAPCs represent a convenient platform to monitor CD4^+^ T cell responses in patients against allergens, autoantigens, or infectious antigens, as it was shown with the antigen ADAMTS13 involved in acquired thrombotic thrombocytopenic purpura (121). In addition, since NIH/3T3-derived AAPCs are able to internalize and process whole protein antigens, and to present derived T cell epitopes, they could be useful to characterize new epitopes of therapeutic interest by mass spectrometry after HLA molecule immunoprecipitation and peptide elution.

## Conclusion and Perspectives

CD4^+^ T cells orchestrate a broad range of immune responses. These last years, our understanding of the CD4^+^ T cell biology has largely progressed but remains incomplete. Indeed, diversity of CD4^+^ cell subsets is more complex than expected, as attested by the discovery of rare functional subsets and reports showing the influence of environmental elements in the plasticity of Th cells and Tregs (3, 14, 122). Understanding the mechanisms underlying functional heterogeneity of CD4^+^ T cells will provide the opportunity to better exploit their properties in protective immunity or in immune tolerance with the ultimate goal of developing potent immunotherapy strategies. Recent studies revealing the roles of Tfh and Th9 subsets in anti-infectious and anti-tumor immunity raise the question of which subtype of Th cells is more adapted to mediate a protective response against specific diseases. In the Treg field, future works are required to characterize the best source of Tregs for immunotherapy by determining relevant markers to distinguish tTregs from iTregs, and carrying out clinical trials to compare clinical benefits of autoantigen-specific Tregs vs. polyclonal Tregs in ACT.

The development of several AAPC systems has led to reproducible and robust expansion protocols for generating high numbers of human antigen-specific CD4^+^ T cells. In addition, some AAPCs could be used under Good Manufacturing Practice conditions and their safety and harmlessness have already been demonstrated in several clinical trials (123–125). AAPCs also have the advantage of being easily customizable by gene transfer to express any costimulatory or co-inhibitory molecule, and are therefore ideal for analyzing all kinds of CD4^+^ T cell responses and providing key knowledges to maximize the success of T cell expansion protocols for ACT.

## Author Contributions

AC, AG, FD, OB, DV, BL-M, J-BL, and OT substantially contributed to the conception of this work, drafted and revised the manuscript, before finally approving its submission. AC, AG, FD, OB, DV, BL-M, J-BL, and OT agreed to be accountable for all aspects of the work.

### Conflict of Interest Statement

The authors declare that the research was conducted in the absence of any commercial or financial relationships that could be construed as a potential conflict of interest.
